# Combining Optical Approaches with Human Inducible Pluripotent Stem Cells in G Protein-Coupled Receptor Drug Screening and Development

**DOI:** 10.3390/biom8040180

**Published:** 2018-12-18

**Authors:** Kyla Bourque, Jace Jones-Tabah, Nourhen Mnasri, Ryan D. Martin, Terence E. Hébert

**Affiliations:** Department of Pharmacology and Therapeutics, McGill University, Room 1303 McIntyre Medical Sciences Building, 3655 Promenade Sir William Osler, Montréal, QC H3G 1Y6, Canada; kyla.bourque@mail.mcgill.ca (K.B.); jace.jones-tabah@mail.mcgill.ca (J.J.-T.); nourhen.mnasri@mail.mcgill.ca (N.M.); ryan.martin@mail.mcgill.ca (R.D.M.)

**Keywords:** single cell approaches, drug discovery, iPSCs, stem cells, imaging

## Abstract

Drug discovery for G protein-coupled receptors (GPCRs) stands at an interesting juncture. Screening programs are slowly moving away from model heterologous cell systems such as human embryonic kidney (HEK) 293 cells to more relevant cellular, tissue and whole animal platforms. Investigators are now developing analytical approaches as means to undertake different aspects of drug discovery by scaling into increasingly more relevant models all the way down to the single cell level. Such approaches include cellular, tissue slice and whole animal models where biosensors that track signaling events and receptor conformational profiles can be used. Here, we review aspects of biosensor-based imaging approaches that might be used in inducible pluripotent stem cell (iPSC) and organoid models, and focus on how such models must be characterized in order to apply them in drug screening.

## 1. Introduction

The last decade has seen a boom in the development of advanced molecular imaging technologies and assay automation which fundamentally transforms the quality and quantity of data that can be generated using cell-based assays. However, the value of such data from a drug development perspective critically depends on the properties of cellular models, and how well they correspond to the physiological systems that they are designed to mimic. For example, cellular functions such as the signal transduction pathways regulated by G protein-coupled receptors (GPCRs) are cell type-specific and may exhibit altered properties depending on “cell context”. Here, cell context refers to the combination of genetic and environmental factors that modulate a given cell’s response to a drug of interest and may include the complement of effector proteins expressed in the cell along with the local microenvironment such as interactions with adhesion proteins or other cells. In this review, we will discuss existing approaches to measuring receptor-driven signaling pathways with a focus on how cell context impacts signaling phenotype. We will then discuss the burgeoning application of inducible pluripotent stem cells (iPSCs) in basic science and drug development, and the factors to consider in the application of iPSCs and their differentiated derivatives to study receptor-driven signaling.

The isolation of embryonic stem cells (ESCs) in 1998 suggested an attractive cellular model for the study of human biology owing to their capacity for self-renewal and differentiation into a large number of distinct cell types [1]. A number of ethical challenges restricted their use as an accessible research model. However, in 2006, Yamanaka and Thomson determined the reprogramming factors required to generate iPSCs from adult somatic cells, side-stepping ethical issues surrounding the use of ESCs and creating a new cellular paradigm for research purposes [2,3,4]. The ability to generate iPSCs has since resulted in significant advances in the biomedical sciences, as researchers can now study human diseases directly from patient-derived cells with applications for both basic understanding of the progression of disease and in approaches to precision and regenerative medicine. With the potential for iPSCs to ultimately be differentiated into any cell lineage, they can provide unlimited sources of untransformed human cells to study the effect of cellular context on protein function. Similarly, iPSCs could provide access to cell types such as neurons and cardiomyocytes for drug screening that were previously unobtainable in large quantities from human sources. Patient-derived iPSCs have been extensively discussed for their potential use in personalized and regenerative medicine, to model patient-specific monogenic disorders, and to generate iPSC and organoid models of complex polygenic diseases [5,6,7,8,9,10]. However, relatively few studies have extensively examined signaling behaviors of iPSC-derived cultures or evaluated the potential for their use in biosensor-based high-throughput drug screening paradigms.

**Figure 1:**
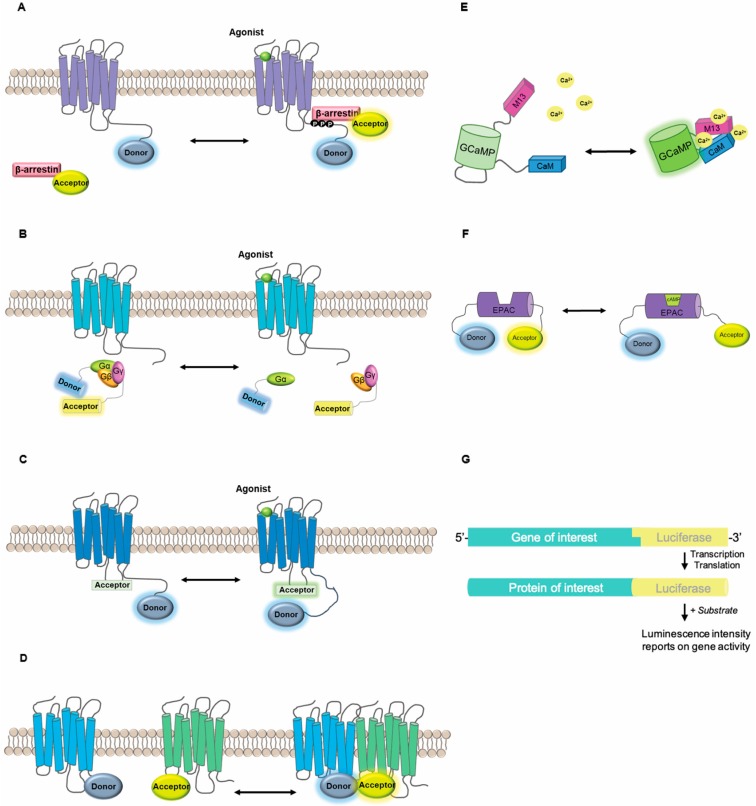
Common intensity, bioluminescent resonance energy transfer (BRET)- and fluorescent resonance energy transfer (FRET)-based biosensors. (**A**) Recruitment-based intermolecular biosensors. In this example, G protein-coupled receptors (GPCR) activation results in β-arrestin recruitment to the phosphorylated receptor. Tagging the receptor of interest with a donor moiety and β-arrestin with a compatible acceptor fluorophore allows us to study the recruitment of arrestins to activated GPCRs. In the absence of agonist, the donor- and acceptor-tagged proteins are far apart and no RET can be detected. Upon agonist induction, β-arrestin is recruited to the receptor at the cell surface resulting in a quantifiable RET event. (**B**) Dissociation-based intermolecular biosensors. GPCR activation results in the dissociation of the heterotrimeric G proteins, Gαβγ; more specifically, the distancing of Gα from Gβγ. Fusion of fluorescent or bioluminescent tags on Gα and on Gγ allow us to study the activation of a GPCR upon agonist stimulation. (**C**) Conformation-sensitive biosensors that report on GPCR conformational dynamics. The highly dynamic nature of GPCRs can be studied by inserting reporter proteins within the coding sequence of the receptor of interest. For instance, fluorophores or motifs specific for fluorescent dyes can be introduced with the intracellular loops of receptors along with a C-terminally fused compatible RET donor. Agonist stimulation can result in the conformational rearrangement in the receptor structure which can be measured by relative changes in the distance/orientation of the chromophores. (**D**) Protein–protein interaction sensors. In order to detect whether two proteins exist in close proximity within the cell, they can be tagged with RET compatible fluorophores, and their interaction can be assayed as described above. (**E**) Intensity-based biosensor GCaMP. This biosensor contains a circularly-permutated fluorophore (GFP) along with the calcium binding domain of calmodulin protein and the M13 domain of myosin light chain kinase. In the absence of calcium, the GFP chromophore is protonated and in a low fluorescence form. In response to calcium binding to the calmodulin domain, a conformational change allows chromophore de-protonation and recovery of fluorescence. (**F**) Conformation-sensitive biosensors that report on intracellular signaling events. These biosensors are synthetic proteins designed to undergo a conformational change in response to interaction with a biological signal of interest such as second messenger binding or phosphorylation of a specific residue. This conformational change results in a change in distance or orientation between a fluorescent or bioluminescent RET pair. In the case of the EPAC biosensor (EPAC or Exchange Protein Directly Activated by cAMP), in the absence of the second messenger cAMP, the two chromophores are in close proximity resulting in a large RET signal. The binding of cAMP leads to a conformational change within EPAC and the corresponding distancing of the two chromophores increases, recorded as a reduction in the RET signal. (**G**) Reporter genes fused to a promoter or gene of interest. Common reporter genes to study the regulation of gene expression include LacZ, GFP and luciferase. Such reporter systems can be used to understand the various factors that are involved in gene expression and to discover potential activators or inhibitors of certain genes/proteins targeted by pharmacological agents.

Genetically-encoded optical biosensors based on fluorescent or bioluminescent proteins have been used extensively to characterize intracellular signaling processes in intact live cells with high spatial and temporal precision (see Figure 1 for in-depth descriptions of commonly used biosensors). The application of such biosensors has perhaps been most widely used in the study of GPCRs. These receptors represent the largest family of mammalian cell surface receptors which have long been considered one of the most druggable targets in the human body. They generally transduce extracellular cues, including peptide hormones and neurotransmitters, by coupling to intracellular signaling partners such as heterotrimeric G proteins (Gαβγ) and β-arrestins. Signal transduction initiated by ligand binding propagates a series of conformational re-arrangements within the receptor structure. Receptor activation may lead to second messenger production, as well as promote the scaffolding of signaling complexes leading to the activation or inhibition of downstream effectors [11]. Biosensors reporting at multiple levels of these signal transduction cascades can reveal that protein behavior at the level of the receptor, and subsequent downstream signaling, are dependent on cellular context. Specific factors known to affect GPCR signaling include the availability and stoichiometry of G protein partners and effectors, direct or indirect interactions with partner receptors in the context of receptor heterodimers, and activity modulation by specific regulators of receptor function and trafficking (reviewed in Ritter et al. [12]). This potential for diversity in the response of specific cells to a drug underscores the need for critical evaluation of cellular models both in basic research and large-scale drug screening.

## 2. Genetically-Encoded Optical Biosensors

### 2.1. Design Considerations

While transduction of many external stimuli relies on a similar repertoire of intracellular signaling molecules, the specific spatiotemporal regulation of signaling events can determine cellular responses [13]. Numerous live cell biosensor assays have been developed as means to decipher signaling downstream of GPCRs and other receptors; for in-depth reviews of biosensor technologies see [14,15,16]. The majority of these biosensors can be classified as follows: (i) intensity vs. resonance energy transfer-based, (ii) bioluminescent vs. fluorescent and (iii) intra- vs. inter-molecular design (Figure 1). Regardless of the specific design parameters, there are three common components: a sensing domain that interacts with a biological target, a measurable indicator (i.e., photons/light), and a mechanism to transduce the sensory event into an optically capturable phenomenon. Intensity-based fluorescent indicators, such as the intracellular calcium indicator, GCaMP, utilize circularly permutated fluorophores which are highly sensitive to the local environment and undergo a change in brightness in response to conformational changes in an associated biosensor domain [17,18,19,20]. Intensity-based bioluminescent indicators such as obelin and aequorin are photoproteins that exhibit substrate-dependent luciferase activity depending on the intracellular concentration of calcium [21]. On the other hand, many biosensors are built on the principle of resonance energy transfer (RET) using either bioluminescent (BRET) or fluorescent (FRET) “donor” moieties which can transfer energy to a suitable “acceptor” fluorophore in a distance- and orientation-dependent manner [22]. Such principles have been exploited to design biosensors reporting changes in protein conformation (intramolecular) or distance between molecules (intermolecular). Intramolecular RET has been used to report conformational changes in proteins of interest including receptors [23,24] and β-arrestins [25], or to generate synthetic reporters that undergo a conformational change when particular signaling events occur, such as binding a second messenger [26] or acting as a substrate for an enzyme [27,28]. Intermolecular RET can be used to report protein movement such as recruitment or disassociation of effector proteins at the plasma membrane, or dissociation of signaling partners such as G proteins from a receptor (Figure 1) [29,30].

Intensity-based versus FRET- and BRET-based approaches each have their own advantages and disadvantages (Table 1). Intensity-based biosensors only require imaging at a single wavelength and tend to have greater brightness and dynamic range, however, comparison between experiments can be challenging due to a strict dependence on expression level of the biosensor in question. On the other hand, RET approaches are measured as an intensity ratio between a donor and acceptor and are quantitatively ratiometric when appropriate controls are applied. Alternatively, FRET can be quantified based on the donor fluorescence lifetime, which only requires the donor fluorescence to be measured and provides more robust indices of FRET efficiency, but this requires specialized equipment [31]. Similarly, BRET relies on the energy transfer from a bioluminescent enzyme such as *Renilla* luciferase (Rluc) to an acceptor such as a protein fluorophore or fluorescent small molecule adjunct [32] (Figure 1). The use of BRET does not require external excitation and thus exhibits low background and avoids the potential confound of autofluorescence, photobleaching or phototoxicity as seen with FRET [33]. However, BRET is generally not compatible with high-resolution microscopy-based imaging due to low photon yield. Numerous efforts have been made to develop improved luciferase enzymes, better suited to bioluminescence imaging, such as Nano-luciferase (Nluc) which produces an intense and sustained luminescence with high signal stability and luminescence efficiency as shown with the Calflux calcium-reporting biosensor [13,34]. Nluc allows luminescence quantification from small numbers of molecules, and is bright enough for single cell BRET imaging applications [13]. For instance, engineering Nluc into a biosensor that reports ERK1/2 activity has shown to improve the sensitivity as well as the temporal resolution of the BRET signals acquired [13].

### 2.2. Biosensor Applications

The design strategies described above have been used to engineer biosensor toolkits to dissect receptor-mediated signaling pathways in precise detail ranging from ligand binding to downstream signal transduction and gene expression. In the past 15 years, numerous receptor-based biosensors have been designed and they have contributed remarkably to our understanding of the basic mechanisms of receptor activation and subsequent signaling. These include intensity-based indicators of receptor activation [35,36,37], intermolecular sensors to study receptor–effector interactions and trafficking [38], as well as conformation-sensitive biosensors capable of revealing different ligand-induced conformational profiles (Figure 1) [23,24,39]. These tools have been critical in demonstrating GPCR activation, trafficking and signaling from intracellular sites [40] and GPCR oligomerization [15,41], as well as biased agonism [39,42]. Assays using BRET and FRET have also been optimized to detect various signaling pathways such as G protein activation or β-arrestin recruitment [38,42,43]. Experiments using full, partial or inverse agonists have shown that different compounds may induce different receptor conformations in live cells [44]. The use of optical biosensors has been important in deciphering the phenomenon of biased agonism or ligand-directed signaling, whereby different ligands may regulate only a subset of pathways downstream of a given receptor [45,46]. Not surprisingly, it has also been shown that cellular context also determines the signaling response to a given ligand.

## 3. How Cell Context Effects Signaling: Evidence from *In Vivo* Studies

Genetically-encoded biosensors can reveal fine spatial and temporal details of cellular signaling processes and have provided a rich understanding of the pluridimensionality of these processes in model systems. However, the utility of such data for predicting therapeutic drug action depends entirely on how well the chosen model reflects the physiological and pathological reality of the disease in question. The application of optical biosensors together with single cell approaches can reveal the granularity of individual cell responses *in vivo* and suggests a link between specific cell states and receptor-mediated signaling over a large population.

### 3.1. Single Cell Sequencing

The expression profile of GPCRs within a specific tissue type is variable and often dynamic in nature. Recent examinations of GPCR expression in primary vascular smooth muscle cells, vascular endothelial cells, T cells and myeloid cells have shown that within a single cell type, GPCR expression profiles are variable and may be altered during development, tissue engineering or in disease states [47,48,49,50,51]. A better understanding of this aspect of cellular context can lead to more efficient drug targeting of cells expressing a disease phenotype. Beyond differential expression of receptors themselves, changes in the stoichiometry or activity of signaling partner proteins such as G proteins can also impact signaling responses. For example, D2 dopamine receptors (D2R) found on medium-spiny neurons of the dorsal and ventral striatum display different sensitivities to dopamine agonists due to differential expression of the Gα subunits Gαi and Gαo [52]. Similarly, in μ-opioid receptor-expressing neurons from dorsal root ganglia, two distinct signaling populations can be delineated based on their responses to morphine, and the difference between signaling groups was dependent on protein kinase C activity [53], suggesting a role for downstream effectors in determining this response. These studies demonstrate that classically defined cell taxonomies based on morphological or incomplete sets of genetic markers may not capture the potential granularity in the signaling behavior of cells which are considered to be a single cell type.

To date, cell type characterization has been dependent on specific cellular behaviors or the expression of relevant molecular markers. Based on the latter, population-based assays have often been performed examining responses in cell populations defined by a set of specific markers. While limitations of this cell type identification criteria were known, the full impact of transcriptomic and functional heterogeneity in cell populations has only recently begun to be appreciated. Through initiatives such as the Allen Brain Atlas, heterogeneous gene expression patterns in the mouse brain have started to be unraveled [54]. With the advent of single cell RNA-seq (scRNA-seq) and the development of more economical methods to pursue this, we are now able to identify cell types through cluster analysis of their individual transcriptomes. These technologies have furthered our understanding of the cell heterogeneity present in organs such as the brain [55,56], pancreas [57], retina [58] and lung [59]. For example, in the visual cortex of an adult male mouse, 49 transcriptomic cell types were identified which included 23 GABAergic and 19 glutaminergic neurons, as well as 7 non-neuronal cell types [56]. These different transcriptomic profiles were linked to distinct cellular phenotypes, as characterized by electrophysiological properties and axon projections. Furthermore, tissues are not only more heterogeneous than previously recognized, but cells characterized by established marker-sets do not always have identical responses to stimuli. For example, a small population of bone marrow-derived mouse dendritic cells responded earlier to lipopolysaccharide (LPS) and regulated the subsequent population level response through release of paracrine factors [60]. This suggests that single cell signaling events must be assessed to better characterize the variation in such complex responses. An important aspect to consider as we move to cells differentiated from iPSCs is whether these models can capture the heterogeneous states of a cell type that may exist *in vivo*. A single cell transcriptomic study of iPSC-derived cortical neurons found that similar to primary cortical neurons, these cells largely expressed genes associated with fetal cortical layers, and 70% of differentiated cells expressed standard markers associated with a specific cortical layer, while a minority co-expressed markers associated with multiple layers [61]. These findings are intriguing because they demonstrate iPSCs exposed to the same differentiation factors are capable of differentiating into multiple cell subtypes within the same population. Similarly, upon differentiation, iPSC-derived endothelial cells exhibited four distinct subpopulations enriched for specific biological functions, such as arterial, activated or immune-responsive [62]. This suggests that iPSCs may have the capacity to recapitulate the heterogeneity of cell populations to be used for modelling various diseases, although further work is required to more fully examine this, including the development of robust and reproducible differentiation protocols, including those which facilitate maturation of stem cells into adult cell types relevant to disease phenotypes [63,64,65].

### 3.2. Single Cell Signaling Assays

For a long time, only patch clamp electrophysiology allowing measures of single channels in individual cells could be used to study the workings of individual proteins. Now, several approaches have been developed that allow us to probe the activity of single proteins in individual cells using optical approaches. Methods using FRET as described above are particularly amenable to use in single cells using intravital confocal microscopy or TIRF (total internal fluorescence reflection) microscopy [51,66,67]. A variety of tools are now available that allow investigation of receptor signaling at the single cell level in live animals *in vivo*. Transgenic animals have been generated expressing the calcium sensor GCaMP [68] and FRET-based reporters of cAMP [69], PKA, ERK1/2 [70,71] and RhoA [72]. Combining these animal models with 2-photon imaging and intravital microscopy techniques has allowed real time analysis of cell signaling with single cell resolution and provides an unparalleled view into how drugs modulate signaling to exert therapeutic or adverse effects *in vivo*. Intravital imaging in mice expressing a RhoA FRET biosensor was used to understand the precise timeframe of RhoA inhibition following treatment with anti-metastatic drugs, information that can be used to inform dosing regimens in clinical practice [72]. Similarly, 2-photon imaging of a PKA (protein kinase A) reporter in tumor endothelial cells revealed low PKA activity which was related to increased permeability, and that this could be reversed using vascular endothelial growth factor (VEGF) receptor inhibitors [71]. While VEGF was found to inhibit PKA activation *in vivo* and in primary cells derived from the tumor site, when the same assay was performed in cultured human umbilical cord endothelial cells, VEGF had the opposite effect on PKA activity, highlighting the importance of assays being performed in the appropriate cell context.

Evidence from *in vivo* genetic and optical techniques has begun to give us a clearer picture of tissue function at the single cell level. In particular, these studies highlight the potential importance of heterogeneity in cellular populations in shaping their responses. A more comprehensive understanding of the role of different cell populations defined by either genetic or phenotypic criteria will guide the development and transition to more physiologically relevant cell-based models. This will not be a simple task but is critical moving forward. It is clear that single-cell “omics” approaches [73] will impact the development of *in cellulo* screens that impact our ability to capture functional heterogeneity as a factor in determining whether or not drug candidates work.

## 4. Incorporating Cell Context into Screening Approaches

Immortalized cell lines such as human embryonic kidney cells (HEK 293) have been widely used because of their ease of culture and proliferation capacity. Cell lines such as HEK 293 cells express numerous receptors, as well as G proteins and downstream effectors as revealed by transcriptome analysis [74] and have traditionally been used as a generic model system to study GPCR signaling. However, to a large extent, this research relies on heterologous expression of proteins of interest, and HEK 293 cells and other immortalized cell lines may not necessarily express the proteomes relevant to cells *in situ*. Ectopic expression of a receptor into such “generic” cellular contexts may not recapitulate the relevant complement of signaling molecules found *in vivo*. Not only are immortalized cell lines often unrepresentative of particular cell types of interest, they often contain large chromosomal aberrations from extended passages, and cells of the same “cell line” have been shown via transcriptomic analyses to vary significantly after time in culture [unpublished data, Hébert lab and colleagues]. As much as the HEK 293 cell has led to advances in several aspects of receptor biology, we are beginning to understand the limitations to this and similar cellular models and moving forward, cell-specific effects on receptor activity should no longer be overlooked. Since numerous cell types can be isolated from animal sources, this offers an alternative tool to explore how cellular context influences signaling signatures. However, primary cells isolated from animals are limited to small-scale studies due to their associated costs and since they do not provide enough cells to be amenable for high-throughput screens. In the search for physiologically- and disease-relevant cells that express the same signalosomes as human tissue, and are amenable to high throughout approaches, inducible pluripotent stem cells may represent a promising alternative. However, before these cells are incorporated into the drug discovery pipeline, their biology will need to be thoroughly characterized.

### 4.1. Inducible Pluripotent Stem Cells and their Differentiated Derivatives: Gene Expression and Functional Validation

As discussed above, cellular responses are traceable to the cause of cell context and depend on the complement of proteins expressed in a given cell. This will have ramifications in drug development bringing back the idea that cellular assays require models that better capture human physiology and disease. For iPSCs to be useful cellular models in drug research their gene expression patterns should match that of the human tissue they are modelling. For example, for differentiated iPSCs to be reliable models in drug discovery, they must express both the therapeutic targets and the relevant microenvironment. Most GPCRs interact with a wide array of different biological partners, including G proteins, receptor dimer partners, effector proteins and lipids, all of which influence receptor activity, downstream signaling consequences and phenotypic outcomes [75,76]. While studies investigating these properties in iPSCs have found promising results, most have been limited in their scope. For example, iPSC-derived cardiomyocytes express several therapeutically-relevant ion channels and GPCRs, such as the α- and β-adrenergic receptors and the muscarinic m3 receptor [77]. Studies also revealed that the gene expression profile of ion channels of control iPSC-derived cardiomyocytes (iPSC-CM) or cardiac disease-specific iPSC-cardiomyocytes matched that of adult left ventricular tissue showing that iPSC-CM are reliable surrogates [78]. Similarly, iPSC-derived dopaminergic neurons endogenously express dopamine D2, amine and 5-HT receptors which represent important targets for antipsychotics [79]. These studies have so far shown that specific targets of interest are endogenously expressed in iPSC-based cellular models, however, large-scale transcriptomic comparisons between iPSC-derived cells and primary tissues are still lacking.

Gene expression studies are often conducted on pooled samples and investigate the average expression profiles of a population of cells. Many research teams have now published their own iPSC differentiation protocols, and evaluating the heterogeneity of differentiated cells at the single cell level would help understand the level of diversity that exists in such populations of derived cells. Single-cell RNA-seq can help shed light on the heterogeneity of iPSC-differentiated derivatives, as was described previously for primary cells. To our knowledge, it seems that most published papers in the literature have focused on a select few genes to validate differentiation procedures and show the coordinated loss of pluripotency markers [80,81]. In order to fully realize the potential of iPSCs as models for drug development we need to uncover the repertoire of GPCR, ion channels and effector proteins present in iPSC derivatives after differentiation. Single cell analyses in primary cells have shown the presence of functionally and pathologically relevant receptor subpopulations where the activation of certain receptors is initially subject to regulation upon inflammatory activation [47,48]. Similar in-depth characterizations as done with primary cells should be undertaken for iPSCs at the genetic and functional level.

In addition to gene expression patterns, many studies have focused on characterizing the electrophysiological properties of iPSC-derived cells. As an alternative to classical electrophysiology, fluorescent indicators such as the Fluor-4-AM calcium sensing dye or FluoVolt voltage-sensitive dye can be used to measure electrophysiological properties and are more amenable to high-throughput approaches. Such dyes have been used to demonstrate that iPSC-derived astrocytes evoked calcium signaling in response to ATP, glutamate, nicotine and acetylcholine [82]. Similarly, FluoVolt has been used to characterize the electrical activity of iPSC-derived cardiomyocytes [83,84]. However, optical dyes suffer from a number of drawbacks, such as potential interference with the biological function of cells, phototoxicity, and lack of available dyes to report diverse signaling pathways. Genetically-encoded biosensors offer an attractive alternative, and a toolkit of genetically-encoded calcium and voltage indicators are now available [85]. Rhesus monkey-derived iPSCs stably expressing the calcium indicator GCaMP6 were generated by introducing the calcium sensor into the AAVS1 safe harbor site [86]. These cells were differentiated into cardiomyocytes whereupon stimulation with the βAR agonist isoproterenol increased the frequency of calcium transients which was reduced by a calcium channel blocker, verapamil [86]. Evaluating the intracellular signaling properties of iPSC-derived cells has so far not received the same attention but can be addressed using similar techniques. Signaling biosensors monitoring different endpoints (Ca^2+^, cAMP, PKA, ERK1/2, etc.) can be expressed using a variety of techniques such as insertion into safe harbor sites or viral transduction. This will not only help better understand signaling pathways downstream of receptor activation but could be used in ligand-directed phenotypic drug screening.

### 4.2. Application of Inducible Pluripotent Stem Cells for Screening Purposes

Drug screening in the age of iPSCs should help improve the attrition rate in drug development given that cell-based assays in these cells hold the potential to better reflect human physiology and disease. This should lead to the development of superior and safer lead compounds by facilitating an additional level of validation before animal studies and clinical trials. Screening using iPSCs has already proven effective in the case of genetically-linked diseases. A large-scale small molecule screen in iPSC-derived neural crest precursors was successful in identifying a compound that rescued the expression of the IKAP protein involved in familial dysautonomia [87]. Their screening method was based on a quantitative reverse transcription PCR assay where they evaluated how small molecules were able to increase expression of either the wildtype and splice variant mutant of the *IKBKAP* gene [87]. They found a role for the α2-adrenergic receptor in regulating *IKBKAP* expression and were also able to replicate their findings in more than one patient-derived clone [87]. Likewise, a phenotypic screen using iPSC derived cardiomyocytes from patients burdened with hypertrophic cardiomyopathies lead to the identification of a compound that was shown to reduce myocyte size [88]. Considering that dysregulated calcium signaling is involved in the hypertrophic process, they also performed calcium imaging studies using a fluorescent dye and demonstrated that this compound in addition to the morphological changes, also reduced intracellular calcium concentration [88]. This study elegantly showed the potential of iPSCs for screening purposes. Screens of this type are slowly gaining traction as phenotypic screens have also been done in iPSC-derived neurons for pain studies and in Alzheimer’s [89,90]. Screens investigating compound toxicity rather than efficacy are also readily feasible with iPSC-based model systems. Population wide effects are often only revealed once a drug is put on the market. Using a collection of hundreds or potentially even thousands of donor cells each with conserved genetic identity, it would be possible to screen for population level effects in an *in vitro* system. In a library of iPSC-cardiomyocytes, it was shown that patient-derived cells from individuals suffering from either hereditary long QT syndrome (an arrhythmia prolonging the QT interval on electrocardiograms), familial hypertrophic or dilated cardiomyopathies were more susceptible to cardiotoxicities than cells originating from healthy individuals. They thus revealed the presence of high-risk sub-populations and emphasized the potential use of iPSC-derived cells for toxicity screens [78].

Cells in a tissue are generally interconnected as they exist in intricate biological networks and communicate via proximal and distal signal transduction mechanisms. Organoids and three-dimensional culture techniques allow exploration of how cells form such complex networks and are thus excellent models to bridge the gap to understand how the local environment, such as extracellular matrix, cell-cell adhesions or paracrine signaling interplay with drug-induced signaling to produce a given effect. Studies utilizing cancer cell lines in a three-dimensional organization revealed the relevance of tumor microenvironment in regard to drug screening. In a colon cancer model, it was shown that cells grown in 3D culture exhibited differential gene expression, namely in genes involved with proliferation compared to cells grown as a monolayer [91]. The authors found that the expression of the epithelial growth factor (EGF) receptor was lower in 3D versus 2D cultures and tested EGFR tyrosine kinase inhibitors, and found that they were less efficacious in 3D than 2D cultures [91]. This study thus revealed that culturing cells in a more physiological environment not only affected their gene expression pattern but also impacts targeted pharmacological responses [91]. Another study produced organoids from primary liver cancer cells and demonstrated that the “tumoroids” retained histological and gene expression patterns similar to the tumor of origin and identified ERK1/2 inhibitors as a potential therapeutic approach [92]. However, obtaining human specimens for all cell types is not always feasible, bringing iPSC-derived organoids to the forefront. One study showed that iPSC-derived cholangiocyte organoids faithfully recapitulated *in vivo* sensitivity to agents that increase or decrease cyst size in polycystic liver disease [93]. The latter arises from invading and proliferating cholangiocytes leading to cyst formation, which prevents normal liver function. This suggests the usefulness of organoids in small-scale drug development paradigms because they appear to replicate *in vivo* effects of known therapeutic agents. Significant progress has also been made generating brain organoids shown to express several different cell types. One study revealed this cellular diversity and demonstrated that such cells form functional and spontaneously active neuronal networks [94]. Organoids derived from iPSCs originating from individuals suffering from the familial form of Alzheimer’s disease recapitulated the hallmarks of AD, including Aβ and tau pathology [95]. The organoids were also sensitive to pharmacological treatment of β- and γ-secretase inhibitors, demonstrating that such structures are able to recapitulate the disease in a dish with the potential for use in the discovery of new drug therapies [95]. Although the majority of organoid research using iPSCs has so far focused on their ability to model developmental processes, and their potential in regenerative medicine, they could also serve as powerful tools in drug development. Just as receptor signaling is shaped by cell context, it will also be affected by the extracellular environment, and culturing cells in more physiologically relevant 3D conditions will lead to better models of human disease. Biosensor expression in organoids combined with imaging technologies like 2-photon confocal microscopy developed for functional fluorescence imaging *in vivo* will allow the characterization of cell signaling from intact organoids, a necessary step in validating organoid models as surrogates for primary tissues in drug evaluation or screening.

## 5. Considerations When Studying Human Disease in Inducible Pluripotent Stem Cell-Based Cellular Models

Before iPSCs can be confirmed as robust models of human disease with high translatability in drug discovery, some concerns need to be addressed, including those that arise regarding whether the epigenetic integrity of individual donors and cell types is altered as a direct consequence of the reprogramming techniques. Recent work has indicated that individual genetic signatures are relatively conserved by demonstrating that numerous genes have more inter- rather than intra-patient variation after reprogramming and differentiation into cardiomyocytes [96]. RNA sequencing data has also shown that the variation observed in iPSCs is a consequence of the transcriptome of individuals rather than other sources, such as memory of the somatic tissue of origin [97]. However, numerous groups have implicated epigenetic modifiers as important factors that influence the reprogramming of somatic cells into iPSCs and their impact should not be ignored (reviewed in [98,99]). While considering iPSC based cellular models for studying human disease, variability in the differentiated cell populations, including batch-to-batch variation and unwanted impurities such as non-differentiated cells, can limit their applicability. Establishing homogenous populations of the desired cell type is thus highly important for meaningful conclusions to be drawn from such studies. Many questions also arise concerning the maturity of iPSC-derived cells, i.e., how well they reflect the functional, genomic and morphological aspects of their counterparts in adult animals or patients. This must be addressed before translating results into the clinic, especially when modeling late-onset diseases. All things considered, the iPSC field is still young but holds tremendous hope to better understand human biology with the promise of personalized medicine. Such considerations could eventually lead to biobanks of stem cell lines derived from different populations that could even be used for clinical trials “in a dish” as another preclinical drug development stage [100].

## 6. Conclusions

**Figure 2:**
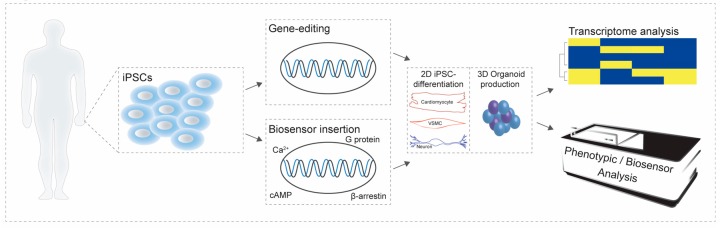
Schematic of biosensor applications within inducible pluripotent stem cells (iPSCs) and organoid models. Adult somatic cells from healthy or diseased individuals can be reprogrammed to generate iPSCs. The genome of the iPSCs can be edited to correct disease-causing mutations or to insert a biosensor via viral delivery or introduction in a safe harbor site. iPSCs can then be differentiated into desired cell-types or to generate organoid models of human disease (Figure 2). Extensive characterization of iPSCs is required, both at the genetic and functional levels, a necessary step for the validation of such models. Next, biosensor expression in iPSC-differentiated derivatives or organoids combined with imaging approaches will allow the characterization of cell signaling to better discern signal transduction events using physiological and disease-relevant cellular models. Understanding the heterogeneity of iPSCs using transcriptomic technologies will also help further validate them as cellular models with high translational potential.

There is significant practicality in utilizing genetically-encoded biosensors in iPSCs and their differentiated derivatives. As discussed above, many studies have taken advantage of optical-based approaches to phenotypically validate differentiated cells. For instance, intracellular calcium is a second messenger in numerous signaling cascades in diverse cells types ranging from neurons to cardiomyocytes. Genetically-encoded biosensors such as the calcium indicator, GCaMP, have been utilized to validate iPSC-CM as well as for proof-of-principle to demonstrate their utility in reporting drug-induced toxicity [101,102]. The amenability of high-throughput screens using genetically-encoded biosensors not only enables validation of differentiation protocols but also can be used for drug discovery. The effect of different types of agonists can be studied in models based on healthy and diseased patients. Such genetically-encoded biosensors can provide us with mechanistic insights into the intracellular signaling pathways that drive disease phenotypes. Expressing biosensors in iPSCs or organoid structures will allow us to acquire knowledge regarding signaling in patient-derived cell populations (Figure 2). Whether iPSCs are used or other sources of stem cells, or even primary cells, the utility of biosensor-based approaches will have to be driven on a case-by-case basis by phenotypic, functional and transcriptomic analyses that capture cellular heterogeneity.

## Figures and Tables

**Table 1:** Advantages and disadvantages of RET techniques- bioluminescence versus fluorescence resonance energy transfer-based sensors. (This has been extensively reviewed in Kauk et al. [14]).

	BRET	FRET
**Advantages**	■ Does not require external excitation ■ Low background resulting in a higher signal-to-noise ratio ■ Simpler instrumentation and quantification of results ■ Better approach for high-throughput screening applications	■ High spatial and temporal resolution ■ Suitable for single cell, subcellular compartments or single protein imaging
**Disadvantages**	■ Lower spatial and temporal resolution because of the low intensity of emitted light by luciferase, excluding Nano-BRET approaches).	■ Requires external light source for donor excitation ■ Lower signal to-noise-ratio due to autofluorescence ■ Potential of photobleaching over time ■ FLIM (fluorescence lifetime imaging microscopy) methods require more complex and expensive instrumentation ■ Potential for cross-excitation of the acceptor and bleed-through effects because of the spectral overlap of fluorophores
